# “I Am the Home Care Agency”: The Dementia Family Caregiver Experience Managing Paid Care in the Home

**DOI:** 10.3390/ijerph19031311

**Published:** 2022-01-25

**Authors:** Jennifer M. Reckrey, Deborah Watman, Emma K. Tsui, Emily Franzosa, Sasha Perez, Chanee D. Fabius, Katherine A. Ornstein

**Affiliations:** 1 Department of Geriatrics and Palliative Medicine, Icahn School of Medicine at Mount Sinai, One Gustave L. Levy Place, Box 1216, New York, NY 10029, USA; 2 Department of Geriatrics and Palliative Medicine, Icahn School of Medicine at Mount Sinai, One Gustave L. Levy Place, Box 1070, New York, NY 10029, USA; Deborah.watman@mssm.edu (D.W.); Sasha.perez@mssm.edu (S.P.); 3 Department of Community Health & Social Sciences, CUNY Graduate School of Public Health and Policy, 55 W. 55th St., New York, NY 10027, USA; Emma.Tsui@sph.cuny.edu; 4 Geriatrics Research, Education, and Clinical Center, James J Peters Veterans Affairs Medical Center, 130 W. Kingsbridge Road, Bronx, NY 10468, USA; emily.franzosa@va.gov; 5 Department of Health Policy and Management, Johns Hopkins Bloomberg School of Public Health, 615 N. Wolfe Street, Baltimore, MD 21205, USA; cfabius1@jhu.edu; 6 Department of Geriatrics and Palliative Medicine, Institute for Translational Epidemiology, Icahn School of Medicine at Mount Sinai, One Gustave L. Levy Place, Box 1070, New York, NY 10029, USA; katherine.ornstein@mssm.edu

**Keywords:** family caregiving, paid caregiving, home care workers, caregiver burden, home and community-based services

## Abstract

As the locus of long-term care in the United States shifts from institutions to the community, paid caregivers (i.e., home health aides, personal care attendants) are providing more hands-on care to persons with dementia living at home. Yet, little is known about how family caregivers engage with paid caregivers. We conducted in-depth, semi-structured interviews (*n* = 15) with family caregivers, of persons living at home with severe dementia, and enriched our findings with data from a second cohort of family caregivers of persons with dementia (*n* = 9). Whether paid caregivers were hired privately or employed via a Medicaid-funded agency, family caregivers reported that they needed to manage paid caregivers in the home. Core management tasks were day-to-day monitoring and relationship building with family caregivers; training paid caregivers and coordinating care with homecare agencies was also described. In order to support family caregivers of individuals with dementia at home, it is important consider their preferences and skills in order to effectively manage paid caregivers. Support of efforts to build a high-quality paid caregiving workforce has the potential to improve not only care delivered to persons with dementia, but the experiences of their family caregivers.

## 1. Introduction

Many persons with dementia opt to remain living in the community with home and community-based long-term services and support, even as their illness progresses [1]. While family caregivers provide the bulk of needed care for persons with dementia living at home, they frequently turn to paid caregivers (e.g., home health aides, personal care attendants, and other home care workers) to provide additional support [2,3]. Demographic shifts towards a shrinking population of younger people, many of whom live at a distance from their older relatives, will only increase reliance on paid caregivers to provide care for persons with dementia living at home [4,5].

According to national estimates, around 10% of persons with dementia living in the community in the United States receive paid care and among those with severe dementia nearly 50% receive paid care [6]. However, in the United States, home and community- based long-term services and support are not routinely covered by health insurance providers or government programs. Instead, paid care in the home is funded by a patchwork of payers including private payers, long-term care insurance, and Medicaid (i.e., insurance coverage primarily for low-income Americans that is administered at the state level) [7]. Within these payer groups, paid care delivery can be structured in a variety of ways: those paying privately for care may hire paid caregivers directly or via homecare agencies, and coverage for Medicaid-funded paid care varies significantly state-by-state and includes both agency-based paid caregivers and those hired directly by care-recipients through consumer-directed care programs [8]. Variability in these financial and care-delivery factors, along with other considerations such as care needs and individual and family preference, contribute to heterogeneous family and paid care arrangements for persons with dementia.

Paid caregivers in the home frequently not only provide hands-on care, but also engage in a wide variety of tasks that support the physical health and social and emotional well-being of those they care for [9,10]. While paid caregivers routinely develop relationships with the family caregivers with whom they share care [11,12,13], such relationships may become even more important when care recipients have dementia [6,14]. A small but growing body of evidence suggests that paid care may have a direct impact on the family caregiving experience and help alleviate some of the burdens of family caregiving [15,16]. Yet despite family and paid caregivers’ collaboration within the home-based dementia care team [16], little is known about how paid care impacts the day-to-day experiences of family caregivers of persons with dementia.

In order to understand family caregiver experiences of working with paid caregivers, we conducted semi-structured interviews with family caregivers of persons living at home with moderate or severe dementia (e.g., Alzheimer’s disease, vascular dementia, mixed dementia). While interview guides focused on the impact of paid care on individuals and families, we identified an additional and emergent theme: the family caregiver experience of managing paid caregivers in the home. This managerial role was distinct from dementia-care tasks performed by either family or paid caregivers and represented a unique responsibility experienced by family caregivers when paid care was present. Because these themes were not an explicit focus of this study but rather emerged in the course of analysis, we analyzed additional interview data from a second study in order to assess the trustworthiness [17] and enrich the content of our findings.

The goal of this analysis is to: (1) describe family caregiver experiences of managing paid care in the home, and (2) describe specific responsibilities related to the management of paid care by family caregivers. Understanding paid caregivers’ experiences managing paid care offers important insights into ways of supporting family caregivers in the difficult task of providing home-based care to their family members with dementia.

## 2. Materials and Methods

### 2.1. Study Setting and Participants

Our research team conducted two distinct but related studies that included qualitative interviews with family caregivers of persons with dementia. In Study 1, interviews were conducted between October 2020 and December 2020 with family caregivers of persons living with severe dementia enrolled in a large, academic home-based primary care program in New York City (*n* = 15). In Study 2, interviews were conducted between January 2021 and August 2021 with family caregivers of people living with primarily moderate dementia recruited via three outpatient geriatric primary care practices in New York City (*n* = 9). Data from Study 2 were used to further explore emergent findings from Study 1.

Recruitment procedures for both studies were similar: we relied on primary care physicians in the target practices to identify family caregivers of individuals with moderate or severe dementia who also received paid care. Specifically, primary care physicians were asked to identify 5–10 of their patients who had: (1) moderate or severe dementia defined as 2 or 3 on the Clinical Dementia Rating scale [18]; (2) a family caregiver age 18 or older who spoke either English or Spanish; and (3) a paid caregiver defined as a paid (non-family) individual providing ongoing care at home regardless of source of payment (i.e., privately paid, Medicaid-funded) or care delivery structure (i.e., private hire, agency-employed). We randomly selected individuals from these pools and, with permission from their primary care physician, approached the individual’s primary family caregiver (as determined by the primary care physician) about an interview.

All study protocols were approved by the Mount Sinai Institutional Review Board (STUDY 19-01206 initially approved 21 September 2021) and participants provided verbal informed consent prior to the interviews.

### 2.2. Data Collection

An interdisciplinary research team with expertise in paid caregiving, family caregiving, home-based care, and qualitative methodology were involved with the conceptualization, recruitment, data collection, and data analysis for both studies. All interview topic areas were informed by previous research and a thorough review of the literature. Each interview began by asking about sociodemographic, functional, and caregiving characteristics for both the person living with dementia and the family caregiver; this information was later used to characterize our study population. Interviews then explored family caregivers’ experience of paid care, although each had a different focus. In Study 1, interviews focused on family caregivers’ experiences and perceptions of the paid caregiver role during the beginning of the COVID-19 pandemic and the impact of paid care on persons with dementia and their families (interview guide included in Supplementary Materials S1). In Study 2, interviews focused on what tasks paid caregivers and family caregivers perform in the home, how these roles are determined, and how paid caregivers fit into the larger care team (interview guide included in Supplementary Materials S2).

The interview guides were piloted for content and clarity with family caregivers not enrolled in the study, and were refined following the first several interviews. Interviews were conducted by either JR (English), DW (English), or SP (English and Spanish) via telephone or via HIPAA-compliant Zoom depending on the participant’s preference. Interviews lasted about 30–40 min. Interviewers wrote memos at the conclusion of each interview capturing emerging themes and interviews were recorded and professionally translated and/or transcribed.

### 2.3. Data Analysis

Data from Study 1 were analyzed in an iterative process using thematic analysis with a combined deductive and inductive approach [19]. The deductive component of the analysis identified themes related to impact of paid care on persons with dementia and their families and the inductive aspect of the analysis identified emergent interview themes. Members of the research team (JR, SP, and DW) independently reviewed several interview transcripts along with post-interview memos and created a preliminary coding scheme, which was revised as codes were applied to the interviews. Two members of the research team (JR and DW) independently applied the final coding framework to the transcripts uploaded in Dedoose qualitative software; discordance in coding was discussed until consensus was achieved [17].

As the team met to review coded data, we identified a distinct theme related to family caregivers’ experience of managing paid care. Four subthemes related to this larger theme (i.e., day-to-day monitoring, building relationships with paid caregivers, training paid caregivers, interacting with agencies) were also identified. These themes were not an explicit focus of Study 1, but rather emerged over the course of analysis and represented a novel and important finding. Given this emergent quality of the themes related to managing paid care, and following existing analyses in which data were blended from studies on related topics [11,20], we sought to build trustworthiness and enrich the depth of our findings [17] by using directed content analysis to explore these themes in a second qualitative study (Study 2) of paid caregiving in dementia (described above) [21]. Specifically, DW reviewed each family caregiver interview transcript from Study 2 and coded text related to the main theme (i.e., family caregiver managing paid care) and the subthemes, with an additional code for emerging subthemes. All coded data was first reviewed with JR in order to understand if and how data from Study 2 complimented data from Study 1; because themes from Study 1 were present in Study 2 and no additional themes emerged, no additional coding was necessary. The findings from both studies were then discussed together with the full research team.

## 3. Results

Table 1 describes the characteristics of family caregivers and persons with dementia in the full sample as well as the subgroups in Study 1 and Study 2. Overall, persons with dementia were on average 90 years old and the majority (83%) were female. The majority (71%) had advanced dementia and required significant ADL support (63% dependent in all ADLs). Family caregivers were typically adult children (71%) and only about 30% lives with their family member with dementia. About two-thirds worked outside the home for pay. Persons with dementia had on average about three paid caregivers, the majority of whom were Medicaid-funded (79%) and agency employed (83%). Most persons with dementia received 24 h paid care (63%).

Both because of the similarities in characteristics between Study 1 and Study 2, as well as the fact that the theme and subthemes related to family caregivers managing paid care that emerged during Study 1 analysis were also present in analysis of data from Study 2, we present data from Study 1 and Study 2 together in the sections that follow. As Table 1 suggests, Study 1 data best illustrates the experiences of family caregivers whose family member had severe dementia and were cared for by paid caregivers 24/7. Data from Study 2 allowed us to further delineate findings for family caregivers of persons with moderate dementia who received less paid care overall; it also allowed us to integrate more perspectives from those who were paying privately for paid care.

### 3.1. Family Caregivers’ Experiences of Managing Paid Care

In interviews with family caregivers, managing paid caregivers in the home emerged as a distinct and consistent aspect of caring for their family members with dementia. This managerial role was distinct from managing the general affairs of the person with dementia (e.g., coordinating appointments, monitoring medical conditions, purchasing needed household supplies) and instead represented a unique responsibility experienced by family caregivers only when paid care was present. The majority of family caregivers described tasks related to managing paid caregivers (described in detail below) without explicitly acknowledging this managerial role; for these family caregivers, managing paid caregivers seemed to be a natural extension of their family caregiving duties and an essential aspect of making sure their family member got the best care possible. “I didn’t know if I should be a part of (this interview) because I don’t physically take care of (my mom) the way (the paid caregivers) do, but my mom is my priority every single day. There’s not a day that I go that I don’t speak to the girls (team of paid caregivers). I speak to the girls two or three times a day to check in to make sure my mom’s okay.” (Study 1, Caregiver 12). This section may be divided by subheadings. It should provide a concise and precise description of the experimental results, their interpretation, and the experimental conclusions that can be drawn.

A subset of family caregivers explicitly referred to themselves as managers. This was most common among family caregivers who directly hired paid caregivers themselves. A son who privately hired his father’s paid caregivers explained, “I am… the home care agency. I personally hire and fire aides, manage their care for her to make sure they are doing what they’re supposed to do.” (Study 2, Caregiver 13). A daughter who hired her mother’s aides from an agency through a consumer-directed care program described her role managing care stating, “It’s just that I’m not putting my mother in a nursing home. That’s my choice and therefore, I have to step up to the plate … The core issues (are) the proper care, the training, picking the right people, watching them. Those things you can do that are important to do if you choose to take care of your parent. That’s part of the deal.” (Study 1, Caregiver 6).

The family caregivers who explicitly described themselves as managing their family member’s paid care frequently described being ill-prepared for this role, which was sometimes described as a job unto itself. This challenge of managing paid care was most pronounced when paid care first started. A daughter described this chaotic time: “I’m very competent… I’m used to balancing balls in the air. But (managing paid caregivers) was all new to me. It was such a different type of job that I was figuring out… For the first six months to a year, it was almost not a day that I wasn’t there (at my mother’s house). That’s the only way I can say, it was by my own judgement, by watching how people interacted with her and with me and with other people. It was just on the job training (for me), really.” (Study 2, Caregiver 7).

### 3.2. Core Management Tasks: Day-to-Day Monitoring and Building Relationships with Paid Caregivers

Regardless of whether or not paid caregivers explicitly described managing their family member’s paid caregivers, most described performing managerial tasks. Two key tasks emerged as core elements of the family caregiver role managing paid care: day-to-day monitoring and building relationships with paid caregivers (Table 2).

Day-to-day monitoring involved tasks like calling paid caregivers on a regular basis to check in, monitoring the home environment with video cameras, reviewing written logs of information about the person with dementia, and conducting unscheduled visits to check in on paid caregivers. In order to build relationships with paid caregivers, family caregivers spent extra time with paid caregivers to get to know them personally, cultivated warm relationships, and were in general attentive to how they treated paid caregivers, to help ensure that the paid caregiver in turn gave their family member with dementia the best care possible (Table 2). These core management tasks were described by caregivers regardless of how paid care was funded (i.e., paid privately, Medicaid-funded) or the structure of care delivery (e.g., directly hired, agency-employed).

Like the experience of managing paid care in general, family caregivers frequently described core management tasks as being more intense when paid caregivers were newly involved in care. As one son described, “After a while, when I see (the paid caregivers) handling my father and he’s good around them and they’re good around him. After a while, there is a familiarity that develops. I have their number and they have my numbers, and they call me up if there’s a concern or a problem.” (Study 2, Caregiver 1). Yet for most family caregivers, both day-to-day monitoring and building relationships with paid caregivers remained important even when paid care was longstanding. As one daughter described. “I’m not a micromanager and I don’t hover over (the paid caregivers). In the beginning I hover, because I want to get the habits engrained. But once I see what they’re doing, I just disappear and I only show up when I need to show up. One thing that I want to throw in there that might help you guys is, get a camera. Get a camera. I use it when I’m away and I want to just see how mom is doing, and then I feel better.” (Study 1, Caregiver 6).

### 3.3. Additional Management Tasks: Training Paid Caregivers and Interacting with Agencies

In addition to the core management tasks of family caregivers managing paid care at home, two additional tasks related to the management of paid care were salient in multiple interviews. The first was the role of family caregivers in training the paid caregivers who worked with their family members with dementia. Whether paid caregivers were hired directly or agency-employed, family caregivers described it as their responsibility to make sure paid caregivers knew how to provide the best possible care and were trained in the unique care needs of their family member. Many caregivers voiced that while paid caregivers had general training in personal care, this wasn’t sufficient to provide the total care that their family members needed; because those with dementia could not advocate for themselves due to their cognitive impairment, it was the family caregiver’s task to provide that additional, individualized training. As one granddaughter described, “I was giving (paid caregivers) the instructions, but… (they) say they know how to do their job. I said, ‘I know you know how to do your job, but this is the patient. You don’t know, so I had to let you know how you’re going to treat my grandma, how to make the food, how to put her comfortable.’” (Study 1, Caregiver 7). Another family caregiver enumerated the things that were not included in the agency’s care plan, but that he had to teach his father’s paid caregivers: “I have to explain what my father is… Things like: he doesn’t eat much… He likes soft food. What else? He has false teeth but he doesn’t like using them for some reason… Sometimes he likes to walk around… he doesn’t like people going in the room, so you’ve got to let him go, follow behind. Don’t let him catch you… Otherwise he’ll get agitated” (Study 2, Caregiver 1).

When paid caregivers were employed by agencies, family caregivers had an additional task of communicating and coordinating with the agency itself. Communications were most frequent early in the course of home care, when families described frequently communicating with the agencies in order to find a paid caregiver that was competent and a good fit for their family member with dementia. A daughter told us, “I did call the agency and let them know that I don’t think it will work out long-term (with this paid caregiver)… I told them I needed someone who’s so mature and knows how to take care of an older person, was more experienced, who understood dementia. I think they based it on that. So the person I have now, the one we got for less than a month—so far so good.” (Study 2, Caregiver 16). However, ongoing communication between family caregivers and agencies was often needed to effectively supervise paid caregivers in the home. For some family caregivers, this was experienced as an additional layer of bureaucracy that made it difficult for those with dementia to get what they needed. As one daughter described, “When it comes to any issues with mama, I try to either get the wound nurse to tell (the paid caregivers) or the aide nurse that comes every six months… Even though that’s my mom, it’s like (the paid caregivers) are working for the agency. They listen to whatever the agency tells them whether it’s good or bad because everybody tries to cover themselves up… (In their view) I’m just the daughter.” (Study 1, Caregiver 1).

## 4. Discussion

When paid care is a component of the home-based dementia care team, family caregivers take on an additional role: managing paid caregivers. Management tasks were common in the full range of usual care arrangements within the United States (e.g., directly hired, agency-employed caregivers) as well as among those with varying dementia severity (e.g., severe dementia requiring 24/7 care, moderate dementia requiring less care). Attending to the experiences of family caregivers as managers of paid care is an important way to support family caregivers in the challenging task of caring for family members with dementia in the home.

The fact that the family caregiver role in managing paid care was common even among paid caregivers employed by agencies speaks to the unique work environment in which home-based paid care occurs. While paid caregivers in the home share many sociodemographic characteristics with direct care workers in other settings such as nursing homes [22], supervision of paid care in the home by homecare agencies is highly variable. For example, over 40% of states do not require licenses for agencies that provide nonmedical personal care (e.g., Medicaid-funded paid care) and therefore these agencies are not subject to paid caregiver training and supervision requirements [23]. Even among paid caregivers employed by agencies subject to state requirements to supervise paid care in the home, supervision is often infrequent or remote, and access to clinical supervisors or real-time support is often limited [10]. More generally, the private and intimate home environment gives paid caregivers a degree of independence and flexibility not present among caregivers working in institutional settings, but also typically leaves them isolated from coworkers and job-based support systems [24,25].

As a result, paid caregivers must frequently learn on the job how to provide the personalized, nuanced care that any individual care recipient needs. When the care-recipients are able to direct care themselves, there may be great variation in how involved family caregivers are in managing care in the home [11,12,26]. However, when care-recipients cannot direct care (as is often the case in dementia and in other settings of advanced serious illness, including care at the end of life) family caregivers must step in to provide additional support and direction. Dementia caregivers in particular are known to provide high levels of care and experience significant caregiver burden [27,28]. Acknowledging the potential challenges of this seemingly ubiquitous management role among dementia family caregiving is the first step so that appropriate supports can be provided [29].

Given the diversity of ways families provide care, it is also important to understand that family preferences for managing paid care are likely to vary and that multiple family caregivers of a single person with dementia may have different preferences. Family caregivers of persons with dementia may experience a wide range of positive effects from providing care and find meaning in providing care [30,31]; being able to engage with paid caregivers to ensure family member are well cared for may be a meaningful component for some caregivers. On the other hand, some family caregivers may prefer to step back from managing paid care but are hesitant to do so because they are unsure of the quality of care their family member will receive otherwise. Our findings on day-to-day monitoring in particular make clear that it takes substantial time for caregivers to trust that paid caregivers are providing good care. Standardized, competency-based training for paid caregivers is an important way to help ensure family caregivers can count on high-quality paid care [23]. Input from family caregivers about the content of that training will prove valuable.

As the ongoing COVID-19 epidemic accelerates the existing shift of long-term care from institutional settings into homes and communities, training paid caregivers to work more effectively with families is essential. At that same time, it will become increasingly important to educate family caregivers to make sure they have the skills to effectively manage paid care. For those who directly hire paid caregivers, frank discussions of mutual expectations for family and paid caregiver roles and responsibilities are an essential first step to meaningful collaboration. It is also important that family caregivers who directly hire paid caregivers understand the historic undervaluation of direct caregivers and strive to pay paid caregivers appropriately for their essential work [32]. Recommendations from organizations like Hand in Hand that support families in ethically hiring and managing paid care in the home should be further disseminated [33].

Education and training for family caregivers working with agency-employed paid caregivers (either privately paid or agency-funded) is also important. Given our findings of the additional challenges family caregivers experience working with agencies, establishing clear and explicit expectations for paid caregiver, family caregiver, and agency roles and responsibilities in care is especially important. As the population of persons with dementia requiring home-based personal care grows, it is important that homecare agencies establish best practices for collaborating with family caregivers and prioritize bi-directional communication with families.

Our findings also point to the larger interdependency of paid and family caregivers in the care of those with dementia living at home. Access to high quality paid care in the home for persons with dementia is a key family caregiving issue. Recent recommendations from the Recognize, Assist, Include, Support, and Engage (RAISE) Act Family Caregiving Advisory Council specifically describe how the paid caregiver workforce shortage impacts the family caregivers who rely on their support: a key report recommendation is to increase and strengthen paid long-term services and support and direct support workforce [34]. In particular, efforts to reduce turnover and promote retention within the paid caregiver workforce will benefit family caregivers whose management work is lessened when stable, trusted paid caregivers are providing care [35,36].

Our project had several limitations. First, themes related to the family caregiver role managing paid care were emergent and, because this topic was not directly explored in the interviews, more nuanced details of what this role entailed may be missing. Second, the family caregivers interviewed all provide care in New York City so their experiences may not reflect the experiences of family caregivers in other places, particularly in those where access to paid care (both privately paid and Medicaid-funded) may be more limited. Finally, perspectives of persons with dementia themselves, paid caregivers, and homecare agencies are not included in this study. Future work should examine multiple perspectives simultaneously to ensure that best practices for family caregivers managing paid care are aligned with the needs and perspectives of all care team members.

## 5. Conclusions

Dementia family caregivers commonly take on roles managing paid caregivers that include tasks like day-to-day monitoring, building relationships with paid caregivers, training paid caregivers, and communicating with agencies. Given the essential role paid caregivers play in the home-based care of those with dementia, focused attention on how to support the family caregivers who manage this paid care may both improve home-based care for those with dementia and make this care more sustainable for their family caregivers.

## Figures and Tables

**Table 1:** Characteristics of Persons Living with Dementia, Their Family Caregivers, and Their Paid Care.

	Full Sample ^a^ (*n* = 24)	Study 1 (*n* = 15)	Study 2 (*n* = 9)
Persons with Dementia			
Age, mean (SD)	89.6 (6.9)	89.1 (8.3)	90.6 (4.1)
Female, %	83	87	78
Latino/a, %	54	67	33
Black, %	8	7	11
Severe dementia, %	71	100	22
Dependent in all ADLs ^b^, %	63	87	22
Family Caregivers			
Age, mean (SD)	60.8 (8.0)	60.7 (9.6)	60.9 (4.4)
Female, %	83	87	78
Hispanic, %	50	60	33
Black, %	8	7	11
Child of Person with Dementia, %	71	60	89
Lives with Person with Dementia, %	29	33	22
Works Outside the home for Pay, %	63	60	67
Paid Care			
Number of Paid Caregivers, mean (SD)	2.9 (1.6)	3.0 (1.3)	2.8 (1.9)
24/7 Paid Care, %	63	73	44
Medicaid-funded, %	79	87	67
Agency-employed, %	83	87	78

^a^ Full Sample includes individuals from Study 1 and Study 2. ^b^ ADL = Activities of Daily Living.

**Table 2:** Core Management Tasks of Family Caregivers Managing Paid Care at Home.

**Day-to-Day Monitoring**
(The paid caregivers) have to know that someone else is looking out constantly for my mother. It involves me constantly calling or visiting or my brother and I visit, making sure everything’s okay in the apartment. Because from experience, if you don’t get extremely involved… (the paid caregivers) might not do their part that they should. You have to just be really on your toes and make sure that they’re doing their part. (Study 1, Caregiver 19)
Some of them, they were falling asleep. I was calling, “Listen. This is not for you to come to sleep. You’re for watching my grandma.” This is one of the problems that I used to have before: they go to sleep. That’s why I put a camera. (Study 1, Caregiver 7)
When (the paid caregivers) say they go out, I believe that they go out… (I know because] I do pop-ups. Sometimes I would go to where they would go, and they would just be there. Or I would call and if they were still at home I would say, “Did you go out today?” They would say, “Oh we didn’t go out today.” (Study 2, Caregiver 16).
**Building Relationships with Paid Caregivers**
I realized I have to work with (the paid caregivers) and make them happy, so when they come in, I give them water. If I get a pizza pie, I share pizza with them. I make it like they’re part of the family. Do you know what I’m saying? I think once you have that rapport with them, then you’ll feel comfortable leaving your mother with them. I can go out and then I know that they’ll do the best that they can do for my mom because I see them as human beings and I understand where they’re coming from. (Study 1, Caregiver 9)
When I go there and I see my mom, I consider these ladies my family now. They take care of my mother. They are my everything when I can’t be there for her… When we go there, we sit around and we talk. We have coffee. We have a familial thing… I wanted my mom’s home to feel like home even though she couldn’t be doing her thing. She couldn’t do anything, but I wanted to walk in and feel that safety. That’s how I feel with the ladies. I feel like they’re part of the family. (Study 1, Caregiver 12)
I find that I have to engage (the paid caregivers) more because I think that they’re more timid to reach out to me for anything, but I constantly reach out to them to reassure them that I like a call if he’s extra “special” today… If there’s anything that they need that I’m not thinking of, you know, house supplies, et cetera. We have a good relationship where they text me if there’s a need or some emergency. (Study 1, Caregiver 20)
It’s important to me that the aide feels that I’m accessible, and that she can tell me if there’s a problem and communicate it to me. That is number one. If that isn’t happening, then that’s bad. (Study 1, Caregiver 4)

## Data Availability

The data presented in this study are available on request from the corresponding author. The data are not publicly available due to the confidentiality of the individuals interviewed.

## Supplementary Materials

The following supporting information can be downloaded at: https://www.mdpi.com/article/10.3390/ijerph19031311/s1, S1: Study 1 Interview Guide, S2: Study 2 Interview Guide.
