# Supplementary material for: “I Am the Home Care Agency”: The Dementia Family Caregiver Experience Managing Paid Care in the Home
Source: Int J Environ Res Public Health. 2022 Jan 25;19(3):1311. doi: 10.3390/ijerph19031311 (PMC8834786; doi:10.3390/ijerph19031311)
Supplement: Supplementary file 1 [file ijerph-19-01311-s001.zip › Supplementary Materials S1 .pdf]

Supplementary Materials S1: Interview Guide Study 1

Thanks for agreeing to take part in this interview. The goal of the interviews is to understand how families and home health aides, home attendants, and other paid caregivers work together to take care of people with dementia and home and how this care changed during COVID. In particular, we're interested in understanding how care provided by home health aides and home attendants matters for patients and families.

I will start by asking you a few questions about your ______ and the care he/she received, and then a few questions about yourself. Then we'll have a more open-ended conversation about if and how this care changed during COVID. Do you have any questions before we get started?

First questions are about your ____.

1. How old is your _____?
2. What is his/her gender?
3. Is he/she Hispanic?
4. What is his/her race?
5. About how many years ago was he/she diagnosed with dementia?
6. Can you please tell me if she/he requires help with each of these tasks?
    a. Walking
    b. Feeding
    c. Dressing
    d. Bathing / showering
    e. Toileting/ using the bathroom
7. Medicaid often helps pay for home health aides or home attendants. Does your ______ have Medicaid?
8. Sometimes people pay for long-term care insurance, which can also help pay for home health aides or home attendants. Does your _____ have long-term care insurance?
9. *If no Medicaid and no LTC insurance confirm:* Does that mean that your _________ pays for his/her health aides or home attendants himself/herself?
10. Do the home health aides or home attendants work for an agency?
11. How many hours of home health aide or home attendant care does he/she receive per day?
12. How many days per week?
13. How many total home health aides or home attendants provide care for your _______?
    a. If multiple, what hours do they work?

Now I want to ask you a few questions about yourself.

1. How old are you?
2. Are you Hispanic?
3. What is your race?
4. Do you live with your __________?

5. Do you work for pay? If yes how many hours per week?
6. How many years have you been helping to care for your _______?

Thank you for all this information. Let's get started with our conversation now.

When the rates of COVID-19 in New York City began to grow in the spring, the care that many people receive from home health aides and home attendants changed. Sometimes these changes were temporary, sometimes the changes were permanent.

1. How did the care that your _____ get from his/her home health aides or home attendants change around this time?
2. Who decided that these changes needed to happen/ that things should stay as they were?
3. *If minimal changes or no changes:* When so many people's changed so much, why do you think your ______ managed to continue more or less uninterrupted? Do you think it is important for your _______ that care continue uninterrupted? If so, why? Can you think of other examples from before COVID when there were changes in paid caregivers and how your _______ reacted?
4. *If significant changes:* How do you think these changes in the person providing care impacted your ____? What is the impact of these changes on you?

For many family members of people living at home with dementia, COVID highlighted the importance of the relationships between family members and home attendants/ home health aides.

5. How would you describe your relationship with your _____'s home health aide(s)? How did these relationships change or develop during COVID?

As I said initially, one of the goals of this interview is to better understand how care provided by home health aides and home attendants matters for patients and families not just in COVID but all the time. I know this has come up in our conversation already a bit, but I wanted to ask you specifically:

6. Can you please tell me more about how the home attendant care that your ______ receives impacts your _______? Can you give examples of where aides positively impacted your _____? Can you give examples of where aides negatively impacted your _____?
7. Tell me more about how the paid care that your ______ receives impacts you. Can you give examples of where aides positively impacted you? Can you give examples of where aides negatively impacted you?

Thank you so much for taking part in this interview. Before we end, I have just one final question. One thing that struck me during the COVID pandemic is home health aides and

home attendants (who people don't usually talk much about) were being praised as "essential workers."

8. In your opinion, what is the most important way home health aides and home attendants impact patients in general? In your opinion, what is the most important way home health aides and home attendants impact families and family caregivers like yourself in general?

Thank you so much for your time! It's very helpful to hear your perspective. I'm going to turn off the recording now.
