# Supplementary material for: “I Am the Home Care Agency”: The Dementia Family Caregiver Experience Managing Paid Care in the Home
Source: Int J Environ Res Public Health. 2022 Jan 25;19(3):1311. doi: 10.3390/ijerph19031311 (PMC8834786; doi:10.3390/ijerph19031311)
Supplement: Supplementary file 1 [file ijerph-19-01311-s001.zip › Supplementary Materials S2.pdf]

Supplementary Materials S2: Interview Guide Study 2

Thanks for agreeing to take part in this interview. The goal of the interviews is to understand what home health aides, home attendants, and other paid caregivers do for older adults with dementia and how they work with families and doctors to keep older adults with dementia healthy at home.

I will start by asking you a few questions about your ______ and the care he/she received, and then a few questions about yourself. Then we'll have a more open-ended conversation your _____'s care.
Do you have any questions before we get started?

I'll start with questions about your ____.

1. How old is your _____?
2. What is his/her gender?
3. Is he/she Hispanic?
4. What is his/her race?
5. About how many years ago was he/she diagnosed with dementia?
6. I'm going to read a list of activities, can you please tell me if she/he requires help with each of these tasks?
    a. IADLS:
        i. Using the telephone
        ii. Travel/ get to places that are beyond walking distance
        iii. Shopping
        iv. Preparing meals
        v. Housework
        vi. House repairs
        vii. Laundry
        viii. Manage medications
        ix. Manage money
    b. ADLS:
        i. Feeding
        ii. Dressing
        iii. Bathing / showering
        iv. Toileting
        v. Transferring out of bed or a chair
        vi. Incontinence
7. Medicaid often helps pay for home health aides or home attendants. Does your ______ have Medicaid?
8. Sometimes people pay for long-term care insurance, which can also help pay for home health aides or home attendants. Does your _____ have long-term care insurance?

9. How many hours of home health aide or home attendant care does he/she receive per day? How many days per week?
10. How many total home health aides or home attendants provide care for your _______?
    a. If multiple, what hours do they work?
11. Do the home health aides or home attendants work for an agency?

Now I want to ask you a few questions about yourself.

1. How old are you?
2. What is your gender?
3. Are you Hispanic?
4. What is your race?
5. Do you live with your __________?
6. Do you work for pay? If yes how many hours per week?
7. How years have you been helping to care for your _______?

Thank you for all this information. Let's get started with our conversation now.

Domain 1: Roles and Role Negotiation

1. People with dementia need help with a lot of different things. This includes daily tasks like bathing, dressing, and cooking that I asked you about before. But it also includes things monitoring for health problems, coordinating care, managing finances, and providing emotional support.
2. To start with, can you describe some of the things that YOU personally do to help your _____?
3. If there are other family or friends who help too, what do THEY do?
4. What does _____'s home health aide or home attendant do?
    a. How has this changed over time?
5. How did you decide who does what? For example, you told me that you do _______, your ____ does _______, and that the home health aide does ____________. How did you decide to set things up this way?
6. How does your relationships with ____'s home health aides or home attendants impact this? For example, sometimes family members only want home health aides or home attendants to do certain tasks for their loved ones after they've worked with them for awhile.
7. *If there is more than one paid caregiver:* Are the roles the same for each home health aide or home attendant? Why or why not?
8. How do aides interact with each other and do some aides play a larger role than others?
9. *If aide through agency:* What are things about the agency's policies that impact this?
10. Are there things you wish your _____ home health aide or home attendant could do or ways you wish she/he could help but can't?
    a. What stops them from helping in this way?

11. Has this division of tasks changed over time? Why or why not?

Domain 2: Larger Care Team

12. Next I want to talk more about other people involved in your _____ ‘s care. We talked about the care that you provide, the care your family provides, and the care your _____’s home health aides or home attendants provide. Many people with dementia also get help from other people. This may include health care providers (like primary care doctors, nurses, physical therapists, and others), those working with insurance or home care agencies (like care managers or coordinators), or those working in the community in other ways (like social service agencies, meal delivery programs, day programs, senior centers, and others). Are there other people who you think play an important role in your _____’s care and keeping him/her living safely at home?
13. Does your _____’s home health aides or home attendants communicate with these other people? Why or why not?
14. Would you go to your ____ doctor if you had a question or concern about an aide? Who would you talk to?
15. What do you consider your _____’s home health aide role in this larger team?

Optional domain 3: BPSD

16. Many people with dementia experience behavioral symptoms related to their dementia. This includes symptoms like delusions, hallucinations, agitation and aggression, depression, anxiety, impulsiveness and inappropriate statements or behaviors, and apathy or indifference. Does your _____ have any of these behaviors or has he/she had them in the past?

Thank you so much for your time! It’s very helpful to hear your perspective. I’m going to turn off the recording now.
